# Effects of Zinc Sources and Levels on Growth Performance, Zinc Status, Expressions of Zinc Transporters, and Zinc Bioavailability in Weaned Piglets

**DOI:** 10.3390/ani11092515

**Published:** 2021-08-26

**Authors:** Xin Ma, Mengqi Qian, Zhiren Yang, Tingting Xu, Xinyan Han

**Affiliations:** 1 Key Laboratory of Animal Nutrition and Feed Science in East China, Ministry of Agriculture, College of Animal Science, Zhejiang University, Hangzhou 310058, China; xinma@zju.edu.cn (X.M.); 22017017@zju.edu.cn (M.Q.); 22017080@zju.edu.cn (Z.Y.); 22017016@zju.edu.cn (T.X.); 2 Hainan Institute of Zhejiang University, Yazhou Bay Science and Technology City, Yazhou District, Sanya 572025, China

**Keywords:** chitosan–zinc chelate, bioavailability, zinc transporter protein, weaned piglets

## Abstract

**Simple Summary:**

Bioavailability of inorganic zinc in animals is low, and large amounts of zinc are excreted into feces, resulting in potential negative impacts on the environment and waste of zinc resources. To reduce zinc supplementation in animal feed, prepared and characterized chitosan–zinc (CS–Zn) chelate was studied to investigate its bioavailability. Dietary CS–Zn improved the weight gain of weaned piglets as compared to ZnSO_4_. The Zn source had a significant influence on the liver, pancreas Zn contents, and the protein expression of ZnT1 and ZIP5 in duodenal mucosa. The Zn contents in the liver and pancreas and the protein expressions of ZnT1 and ZIP5 increased linearly with increases in the added Zn level. Multiple linear regression and the slope-ratio methodology showed that the bioavailability of CS–Zn was 110.9% or 149.0% relative to ZnSO_4_, respectively, using zinc content in the liver or pancreas as the response parameter. These results indicate that CS–Zn shows enhanced bioavailability, suggesting a good potential substitute for inorganic zinc in animal nutrition.

**Abstract:**

The present study was conducted to explore the bioavailability of chitosan–zinc chelate (CS–Zn) in weaned piglets, and its characteristics of prepared and oral safety were also involved. A total of 210 crossbred weaned piglets (Duroc × Landrace × Large White) with a mean body weight of 6.30 kg were randomly assigned into seven dietary treatments involving a 2 × 3 factorial arrangement with two Zn sources (CS–Zn and ZnSO_4_) and three levels of added Zn (50, 100, 150 mg Zn/kg) plus a Zn-unsupplemented control diet. The feeding trial lasted 42 days. The AFM image of CS–Zn showed a rougher appearance and smaller size particles. The changes in spectrum peaks evidenced the successful chelating of Zn^2+^ with chitosan. The XRD patterns revealed the formation of a new crystalline phase. Moreover, the oral acute toxicity test of CS–Zn showed no lethal effects on mice. Weaned piglets fed dietary CS–Zn showed improved weight gain and decreased diarrhea incidence. Additionally, the bioavailability of CS–Zn was higher than that of ZnSO_4_ in piglets. Taken together, these results indicate that the prepared CS–Zn chelate, with rough surface and crystalline phase, is non-toxic and show enhanced bioavailability.

## 1. Introduction

In recent years, heavy metal excretion from pig production has received more and more attention from the public due to its potential negative impacts on the environment and human health. In animal science, zinc is usually supplemented into the feed to improve performance as an inorganic form (ZnO or ZnSO_4_). In order to lessen diarrhea and improve performance in weaned piglets, the common recommendations are 1000–4000 mg kg^−1^ of inorganic Zn [1]. Notice No. 2625 of the Ministry of Agriculture of China stipulates that the supplemental amount of zinc in the form of ZnO in the compound feed of piglets for the first 2 weeks after weaning should not exceed 1600 mg kg^−1^. However, according to the National Research Council (NRC, 2012) [2], the requirement for zinc supplementation in the feed of weaned piglets is 60–100 mg kg^−^^1^. The abusive use of zinc additive in livestock husbandry is still serious, and large amounts of zinc are excreted into feces due to the low bioavailability of ZnO or ZnSO_4_, resulting in soil and river contamination [3] and a huge waste of zinc resources [4]. It is made even more worrying that enteral bacteria in food-producing animals may develop antibiotic resistance due to high dose trace elements supplementation such as zinc, while such bacteria carrying resistance genes may be transferred from animals to humans [5]. Studies have shown that organic zinc is a potential alternative for inorganic zinc to reduce zinc excretion [6]. Therefore, zinc from different sources, which have higher bioavailability, have attracted researchers’ interests.

Chitosan (CS) is a deacetylated derivative of chitin, containing a large number of amine groups and hydroxyl groups. This structure brings tremendous capability to chelate metals and form CS–metal complexes [7]. Therefore, one of the essential properties of chitosan is its capability to chelate powerfully with heavy metal ions, especially with Zn, Cu, Hg, Pb, Cr, and so on. Various previous applications proposed were in water purification for wiping off toxic metal ions or other ions [8]. Chitosan also plays an important role in antibacterial activity and protection against pathogenic infections [9,10]. Recently, several kinds of chitosan derivatives were studied as carriers for therapeutic proteins and on disease therapies [11]. In addition, there are many hybrids, which consist of chitosan and some substance (such as zeolite, granular activated carbon, etc.), could exert better function in varied areas.

Chitosan–Zn chelate (CS–Zn) is a chelate of Zn^2+^ with CS. After CS binding to Zn ions through oxygen, nitrogen, or the combination of them, it will probably have better biological activity [12]. Our previous studies showed that dietary chitosan–zinc chelate could not only reduce post-weaning diarrhea and increase growth performance but also improve the intestinal structure in weaned piglets. Moreover, dietary 100 mg kg^−1^ Zn as CS–Zn was discovered to have similar biological effects to dietary 3000 mg kg^−1^ Zn as ZnO [13], implying that CS–Zn might be a kind of novel source of zinc in pig production.

The evaluation on the characteristics, safety, and bioavailability of CS–Zn chelate is important for its potential applications in food, feed additives, and health care products. Therefore, we hypothesized that dietary CS–Zn would show higher bioavailability and less zinc content of feces in pig production. The present study dealt with (1) the characteristic of prepared CS–Zn through several physical methods including atomic force microscopy (AFM), Fourier transform infrared (FT-IR), and X-ray diffraction (XRD), (2) the oral acute toxicity of CS–Zn on mice, and (3) the assessment of its bioavailability in weaned piglets by using the concentration of zinc in the liver and pancreas as indicators.

## 2. Materials and Methods

### 2.1. Materials

Chitosan, MW 40,000–50,000 and 95% degree of deacetylation, was acquired from Gold Shell biochemical Co. Ltd. (Taizhou, China). ZnSO_4_·7H_2_O, acetic acid, absolute ethyl alcohol, and acetone were analytical grade chemicals. All the reagents were used without further purification.

### 2.2. Preparation of CS–Zn Chelate

The chelate was prepared as follows: Chitosan (3 g) was dissolved in approximately 2 mL acetic acid (AR) by continuously stirring on the magnetic stirrer. During the stirring, an amount of zinc sulphate (v:v = 4:1) was added into the solution. We continued stirring until the mixture was homogeneous. AR and water were added to maintain the pH value around 5. After being stirred for 8 h on the magnetic stirrer, the mixture was poured into absolute ethyl alcohol and acetone solution (1:1). Then, the supernatant was discarded and the white precipitate was washed with distilled water several times until there was no Zn^2+^ in eluent. Finally, the precipitates were kept at 60 °C for about 10 h and, then, cooled to 25 °C.

### 2.3. Characterization

#### 2.3.1. Atomic Force Microscopy Analysis

Surface structure was measured by SPM-9500J3 atomic absorption spectrometry (AFM, Shimadzu Corporation, Kyoto, Japan). Chitosan–Zn chelate was sonicated for 10 min to facilitate dissolution and properly diluted in distilled water. The stock solutions were diluted with 10 mM ammonium bicarbonate buffer (pH 8) to a final concentration of 1–5 μg mL^−1^. Then, 10 microliters samples were drop-deposited onto freshly cleaved sheets of mica. The coated mica preparations were air-dried on a heating block at 37 °C, promoting homogeneous spreading of the sample across the mica, reducing molecular aggregation on drying and subliming off the buffer [14]. After drying, the sample was moved onto the AFM.

#### 2.3.2. Fourier Transform Infrared Analysis

FT-IR spectra were collected in the absorbance mode in the frequency range of 400–4000 cm^−1^ using an AVATAR 370 spectrophotometer (Perkin Elmer, Waltham, MA, USA). The samples were mixed uniformly with potassium bromide in a 1:5 ratio (sample: KBr). The KBr discs were equipped by condensing the mixed powders at a pressure of 5 tons for 5 min in a hydraulic press. The discs were scanned in the parameters of 400–4000 cm^−1^ to acquire FT-IR spectra.

#### 2.3.3. X-ray Diffraction Analysis

Identification of the crystal phases was executed by X-ray diffraction analysis. The XRD patterns were acquired by Bruker-AXS D8 Advance, with Cu Ka radiation (λ = 0.154 nm) at 40 kV and 30 mA. X-ray diffraction data were obtained from 2θ = 5–35° at a scanning rate of 0.5°/s at room temperature.

### 2.4. Oral Acute Toxicity in Mice

Animal testing was approved by the Experimental Animal Center of Zhejiang Province (SCXK 2014-0001). All experimental procedures involving animal care and sampling were performed with compliance of the institutional guidelines for animal research. Fifty ICR mice, weighing 18–22 g, male and female each half were purchased from Shanghai laboratory animal Center (Shanghai, China). The mice were housed by sex and fed on commercial pellet diet, given deionized water ad libitum, and kept in plastic cages in a 21–26 °C controlled temperature, 50–70% relative humidity room. After three-day acclimation, the mice were fasted over night before the experiments and were divided into 5 groups of 5 male and 5 females at random. The animals were treated with each dose level of CS–Zn (2.15, 4.64, 10.0, 21.5, 46.4 g kg^−1^), respectively. Toxins CS–Zn (1.08, 2.32, 5.0, 10.76 g kg^−1^), dissolved in soybean oil to 20 mL, were given to mice by gastric gavage at twice in a volume of 0.2 mL kg^−1^ body weight. While the 46.4 g kg^−1^ group were administrated with CS–Zn (11.6 g in 20 mL soybean oil) at third times. All the mice were weighed before the treatment and after death immediately. After gavage of the toxins, mice were observed daily for 2 weeks and the signs of general health status, toxic symptoms, and mortality were recorded. After two-week treatment, the animals were sacrificed for histopathological examination. The LD_50_ values (lethal dose of the toxins for 50% of the treated mice), based on 24 h mortality data, were calculated at 95% confidence level.

### 2.5. Bioavailability Analysis in Piglets

#### 2.5.1. Animal Treatment

The present study followed the institutional guidelines for the care and use of animals. All the animals and samples designed for the experiment were certified by the Animal Care and Use Committee of Zhejiang University (SYXK 2012-0178).

A total of 210 crossbred weaned piglets (Duroc × Landrace × Large White), aged 21 days and weighing about 6.30 kg, were randomly assigned to 7 dietary treatments, and each treatment was replicated 3 times with 10 piglets per replicate. The group fed a corn-soybean meal basal diet (Table 1) without extra Zn supplementation was applied as control. Compared to the control, the other 6 dietary treatments were fed a basal diet supplemented with 50 mg kg^−1^ of Zn as ZnSO_4_, 100 mg kg^−1^ of Zn as ZnSO_4_, 150 mg kg^−1^ of Zn as ZnSO_4_, 50 mg kg^−1^ of Zn as CS–Zn, 100 mg kg^−1^ of Zn as CS–Zn, and 150 mg kg^−1^ of Zn as CS–Zn, respectively. CS–Zn, a Zn–chitosan chelate compound, was provided by the Zhejiang University Feed Science Institute (Hangzhou, China), and the content of Zn was 16.7%. Basal diet was formulated in accordance with the nutrient requirements recommended for weaned piglets by NRC (2012) [2]. The analyzed Zn contents in diets were presented in Table S1.

The piglets were given free access to feed and water throughout the experiment (42 days). Piglets were weighed at the start and the end of the feeding experiment individually to calculate the average daily gain (ADG). Feed intake was recorded daily per pen to calculate the average daily feed intake (ADFI), then feed to gain ratios (F/G) were calculated for each pen. Mortality was considered in the calculations of ADG and ADFI. Diarrhea incidents, feces consistency, and color were recorded every day. Diarrhea index was scored as follows: 0, normal feces (solid); 1, moist feces (semi-solid); 2, mild diarrhea (loose feces); 3, severe diarrhea (watery feces). Mild diarrhea and severe diarrhea were both considered as diarrhea. The number of weaned piglets suffering from diarrhea and its duration were monitored and recorded during the experiment. The rate of diarrhea was calculated as the total number of weaned piglets with diarrhea multiplied by number of days of diarrhea divided by the total number of piglets multiply duration of feeding experiment.

#### 2.5.2. Sample Collection

At the end of the feeding experiment, 6 piglets (2 piglets per pen) from each dietary treatment were selected randomly to be euthanized after a 12-h fast. Liver and pancreas samples were excised and stored at −80 °C until analysis. Duodenal mucosa was stripped from the seromuscular layer, transferred to Eppendorf tubes, snap-frozen in liquid nitrogen and stored at −80 °C. Fecal samples were collected by using total feces collection method from each treatment on the 40, 41, 42 days, mixed and homogenized with 10% hydrochloric acid and stored in a refrigerator at −20 °C.

#### 2.5.3. Zinc Content and Transporter Analysis

After being wet digested with nitric acid via microwave, the zinc concentrations in the liver, pancreas, and feces were measured by an atomic absorption spectrophotometer (Thermo Scientific M5 AA Spectrometer, Waltham, MA, USA).

The protein expressions of Slc 30 A 1 Zinc Transporter (ZnT1) and Slc 39 A 5 Zinc Transporter (ZIP5) in the intestine were detected by Western blot. The total proteins were extracted by the total protein extractive kit containing protease inhibitor cocktail (Haoji Biotechnology, Hangzhou, China) and, then, detected by a BCA protein quantitative kit (Haoji Biotechnology, Hangzhou, China). Protein was separated by SDS-PAGE and transferred onto PVDF membrane (Millipore, MA, USA). The membranes were closed in 5% non- fat milk powder for 1 h. After several washes, the membranes were incubated with a 1:500 dilution of anti-ZnT1 antibody (Santa cruz SC-27501) or anti-Zip5 antibody (abcam ab76191) at 4 °C overnight. Similarly, after several washes, each membrane was dissolved in 2% non-fat milk powder for 1 h. According to the manufacturer’s protocol (Super Signal), the signals were estimated by West Dura Extended Duration Substrate. In the end, the data were analyzed by Bandscan software system (v5.0) and presented as the mean and standard deviation.

### 2.6. Statistical Analysis

To explore the effect supplemental Zn, data were analyzed by using a single degree of freedom contrast to compare all supplemental Zn treatments with the control. Dates excluding the control were further analyzed as 2 × 3 (source × level) factorial arrangement of treatments by two-way ANOVA using the general linear model (GLM) procedure (IBM SPSS Statistics 20). The model included the main effects of supplemental Zn level and source, as well as their interaction. When an effect was significant, means were compared by the least significant difference (LSD) method to determine specific differences between means. Orthogonal polynomials were applied to judge linear responses of dependent variables to daily intake amount of dietary analyzed Zn. Inputting the concentration of Zinc in the liver, pancreas as dependent variable, and the addition of CS–Zn or ZnSO_4_ as independent variable, the statistical model was as follow: Y = c + aX_1_ + bX_2_, where X_1_ is the addition of CS–Zn in daily diet and X_2_ is the addition of ZnSO_4_ in daily diet. According to the standard of inorganic zinc sulfate (100%), the bioavailability of CS–Zn relative to ZnSO_4_ was calculated by using the multiple linear regression slope ratio method, equaling to a/b*100%. When the probability of an independent variable’s t-value was less than 0.01, the effect of X_1_ and X_2_ on the dependent variable was significant. The square of R was presented as the degree to which the dependent variable is dependent on the independent variable. The collected data were analyzed and expressed as mean ± SD. Statistical significance was determined at *p* < 0.05.

## 3. Results and Discussion

### 3.1. Atomic Force Microscopy Analysis

**Figure 1:**
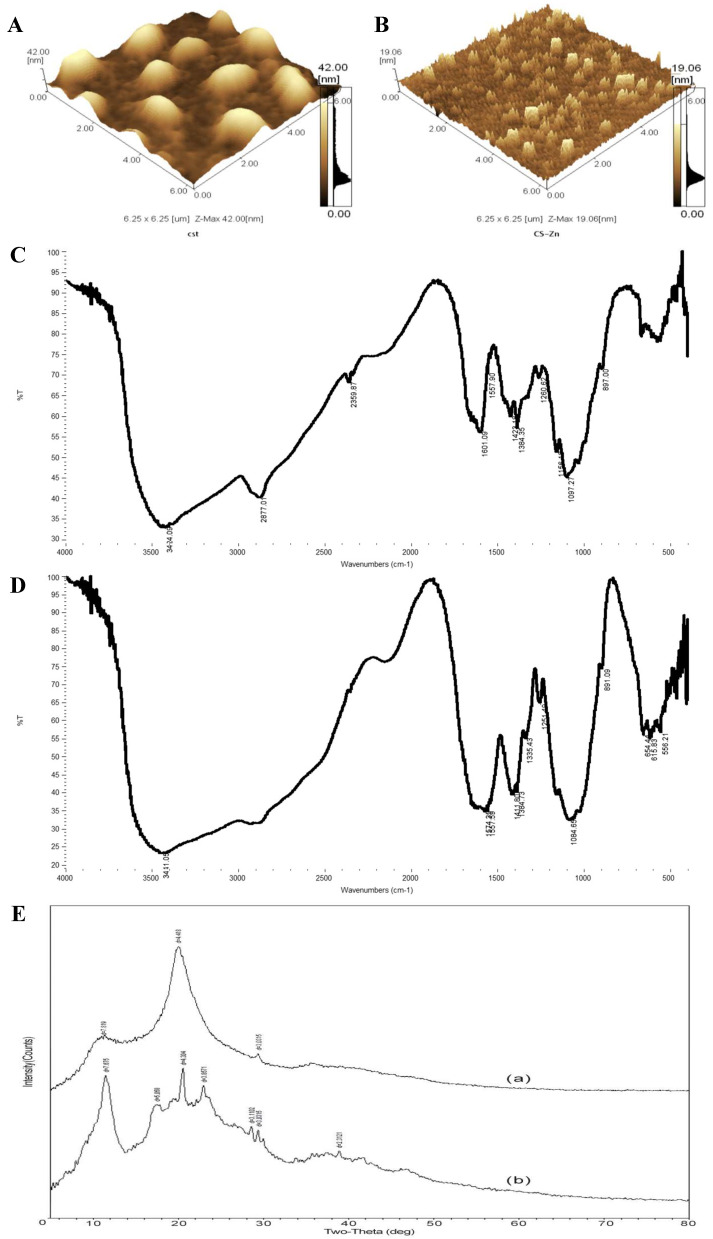
(**A**) AFM images of chitosan. (**B**) AFM images of chitosan–zinc chelate. (**C**) FT-IR spectrum of chitosan. (**D**) FT-IR spectrum of chitosan–zinc chelate. (**E**) X-ray diffractogram of (a) chitosan, (b) chitosan–zinc chelate.

Atomic force microscopy is usually used to exam the surface image. The three-dimensional AFM image of CS and CS–Zn were shown in Figure 1. Figure 1A,B is shown over an area of 6250 nm^−1^. The particles of CS distributed regularly. The morphology of particles was a ball or ellipsoid shape. The surfaces of CS samples had typical hills and valleys morphology with a smooth surface. However, the AFM image of CS–Zn consisted of flakes growing on the entire surface and showed a rougher appearance, which was different from that of CS. Moreover, the size of CS–Zn was smaller than that of CS.

The significant change in the structure of CS–Zn might potentially be the existence of –NH_2_ group and –OH group, as the metal-chelating ligand bonded with mineral impeccably. In other words, metal ions such as Zn^2+^ were very strongly bound to the chitosan.

### 3.2. Fourier Transform Infrared Analysis

FTIR analysis was used to verify the characteristic functional groups on the surface of CS–Zn chelate. The obtained FT-IR spectrum of CS and CS–Zn were shown in Figure 1C,D. Firstly, the FT-IR spectrum of CS–Zn consisted of a number of sharp absorptions consisted with a well-defined molecular structure. Secondly, the FT-IR spectrum of CS–Zn chelate showed some variations from that of CS. The band peak at 3424.09 cm^−1^, corresponding to the multiple absorption peaks, was developed by stretching vibration of the –NH_2_ group and –OH group as well as inter- and intra-molecular hydrogen bonding. However, these peaks became weak and shifted to low frequency, which was as similar as the results reported by Saeed et al. (2014) [15]. These were ascribed to the interaction between chitosan and ions. The asymmetric stretching of CH_3_ and CH_2_ of chitosan polymerat developed a peak at 2359.87 cm^−1^, which was nearly with the results observed by Guo et al. (2005) [16]. However, it disappeared when Zn^2+^ was added, which was consistent with the studies showed by AbdElhady (2012) [17]. The absorb bands at 1601.09 cm^−1^ (a sign of –NH_2_ group and at 1384.35 cm^−1^ (a sign of –OH group) both clearly shifted to a lower wavenumber. These implied that the –OH group and –NH_2_ group were involved in this reaction [18]. Therefore, the successful chelating of Zn^2+^ onto the CS was evidenced by the changes in the peaks of CS–Zn.

### 3.3. X-ray Diffraction Analysis

Figure 1E showed the XRD pattern of the CS (a) and CS–Zn (b). A broad diffraction peak between 10° and 30° was observed in the XRD pattern of the CS–Zn, while the XRD pattern of the CS only showed two characteristic peaks at 10.5° and 21.1°, which were corresponding to d (7.819) and d (4.418), respectively. This result was agreed with the study reported by Li et al. (2010) [19]. However, both of them were weakened, which might be attributed to Zn^2+^ disturbing (or impeding) the order of the polymer chain [18]. Even some imaging observed the second characteristic peak disappeared [20]. Obviously, CS chelated with Zn^2+^ showed more numerous and sharper X-ray diffraction bands than that of untreated CS. This indicated that the complexes obtained by chelation had a single crystalline phase. The pattern was consistent with the study of Wang et al. (2004) [12], revealing the formation of a new crystalline phase.

### 3.4. Oral Acute Toxicity in Mice

The results of oral acute toxicity of CS–Zn were shown in the Table S2. The data indicated that oral administration of CS–Zn would be placed in the category of least concern, “relatively harmless”. It also showed that the death of mice gavaged with CS–Zn occurred starting from the dose of 10.0 g kg^−1^ CS–Zn. Mice treated with each dose (except 2.15 g kg^−1^ group) of the samples were apathetic, piloerection, and clustering in one hour. However, the mice treated with 21.5 g kg^−1^ or 46.4 g kg^−1^ subsequently recovered within 30 min to an apparently normal state. After that, no mortality and symptoms were observed during the two-week experimental period. On the contrary, all the mice treated with the maximum dose (46.4 g kg^−1^) were dead after the second time of gastric gavage. Furthermore, no obvious differences were found in the final body weight of the three treatments. The LD_50_ values (dose causing lethality in 50% of treated mice) for female and male mice were both 10.08 g kg^−1^. These results indicated that there were no differences between female and male mice. The LD_50_ values showed that CS–Zn did not result in pathological symptoms and lethal effects of mice according to the acute toxic classifications of WHO criteria.

### 3.5. Bioavailability Analysis

**Figure 2:**
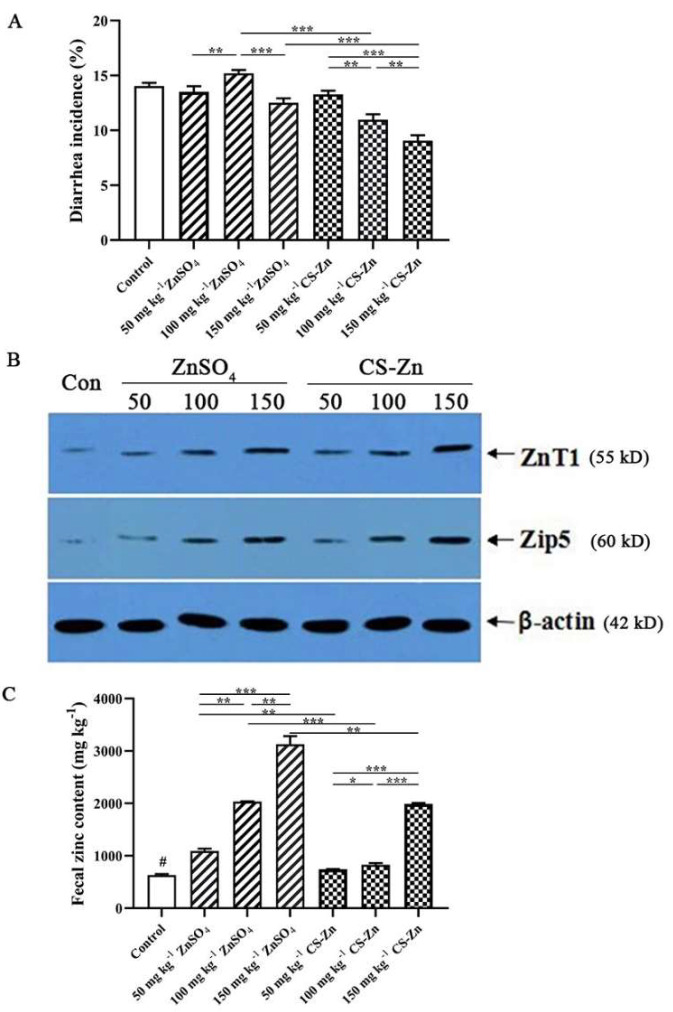
(**A**) Diarrhea incidence of weaned piglets fed dietary CS–Zn or ZnSO_4_. The columns represent means ± SD. (**B**) Intestinal zinc transporters protein expression of weaned piglets fed dietary CS–Zn or ZnSO_4_. The bands are representative blots from one of the six pigs. The relative protein expression was expressed as the ratio of the target protein and β-action. (**C**) The zinc content in feces of weaned piglets fed dietary CS–Zn or ZnSO_4_. The columns represent means ± SD. * Differences were considered significant at *p* < 0.05. ** Differences were considered significant at *p* < 0.01. *** Differences were considered significant at *p* < 0.001. ^“^#^”^ Different from all supplemental Zn groups, *p* < 0.05.

Growth performance of weaned piglets affected by dietary Zn source and level was shown in Table 2. Zn source, added Zn level, and their interaction between Zn source and added Zn level significantly affected ADG, ADFI, and F/G (*p* < 0.014). Compared with the piglet’s diets supplemented with ZnSO_4_, the piglets fed diets supplemented with CS–Zn had higher ADG and ADFI and a lower F/G (*p* < 0.05). The piglets fed diets supplemented with 150 mg kg^−1^ of Zn had higher ADG and ADFI than those fed diets supplemented with either 50 or 100 mg kg^−1^ regardless of Zn source (*p* < 0.01). Diarrhea incidence of weaned piglets fed dietary CS–Zn or ZnSO_4_ is presented in Figure 2A. The interaction between Zn source and added Zn level affected diarrhea incidence significantly. By adding 100 mg kg^−1^ Zn as CS–Zn, 150 mg kg^−1^ Zn as CS–Zn, and 150 mg kg^−1^ Zn as ZnSO_4_, the diarrhea incidence of weaned piglets was obviously decreased, compared with control treatment (*p* < 0.01). Moreover, the piglets receiving 100 mg kg^−1^ Zn as CS–Zn and 150 mg kg^−1^ Zn as CS–Zn had a lower diarrhea incidence than that of the piglets fed the diets containing the same level of ZnSO_4_ (*p* < 0.001). In CS–Zn treatment, the diarrhea incidence was significantly decreased with the increase in Zn level.

In the present study, dietary Zn improved growth performance and decreased diarrhea incidence apparently; moreover, diets supplemented with CS–Zn were better than those supplemented with ZnSO_4_ in general. Buff et al. (2005) [21] reported that dietary supplementation of zinc–polysaccharide improved growth performance in weaned piglets. However, Case and Carlson (2002) [6] found that there was no significant difference in growth performance between the piglets fed 500 mg kg^−1^ of Zn as zinc–amino acid chelate and those of control group. Another study observed that organic zinc either as polysaccharide or proteinate complex had no effect on the growth performance [4]. The differences among the results may relate to the health level of trial animal, gene differential expression, breeding environment, feeding schedule, and especially, to differences in chelating agent. In recent years, domestic and international studies have reported that organic zinc increases the growth performance only in the growth stage, mainly to increase the feed intake [22]. In actual production, the addition of Zn to the diet of weaned piglets has the effect of promoting weight gain. One of the reasons is likely to be generated by participating in the taste element to affect the structure and function of oral mucosal epithelial cells. Moreover, Zn takes part in the synthesis of a variety of metabolic enzymes in the body, improves the digestion function, and enhances appetite. At the weaned stage, piglets suffer from one of the most stressful events, as they are fed with solid diets instead of liquid milk, which results in increased susceptibility to diarrhea. It was reported that zinc ion could inhibit the respiratory chain of pathogenic *Escherichia coli*, leading to diarrhea [22]. The results that CS–Zn reduced the incidence of diarrhea might depend on not only the function of zinc ion but also the antimicrobial activity of chelated chitosan. Another possible reason may be the improvement of the intestinal microflora and the immune function of weaned piglets fed diets containing CS–Zn [13]. The reduction in diarrhea may make a contribution to the improved growth performance.

The effect of dietary Zn source and level on the content of Zn in the liver and pancreas of weaned piglets was shown in Table 3. Compared with the piglets fed the control diet, the piglets fed diets supplemented with Zn had higher Zn contents in the liver and pancreas (*p* < 0.05). The interaction between Zn source and added Zn level affected pancreas Zn content (*p* < 0.001), but had no effect on Zn content in the liver (*p* = 0.732). Zn source and added Zn level significantly affected Zn contents in the liver (*p* < 0.007). The piglets fed diets supplemented with CS–Zn had higher Zn contents in the liver than those fed diets supplemented with ZnSO_4_ (*p* < 0.05). Compared with ZnSO_4_, all levels of CS–Zn remarkably increased the zinc content in pancreas (*p* < 0.05). There were significant differences in the zinc content in pancreas among 50 mg kg^−1^ CS–Zn, 100 mg kg^−1^ CS–Zn, and 150 mg kg^−1^ CS–Zn groups (*p* < 0.05), which was appropriate for ZnSO_4_ groups. The Zn contents in the liver and pancreas increased linearly with added Zn increasing (*p* < 0.001). The effects of dietary Zn source and level on the content of Cu in the liver and pancreas was shown in Table S3. The Zn source and added Zn level had no significant influence on the content of Cu in the liver, but had a significant effect on that in pancreas. The concentrations of pancreatic Cu in 50 mg kg^−1^, 100 mg kg^−1^, and 150 mg kg^−1^ CS–Zn groups and the 150 mg kg^−1^ ZnSO_4_ group were significantly increased, which was similar with the study of Hill et al. (2014) [23] who observed that adding organic zinc can increase the concentration of Cu in kidney. CS–Zn can not only affect the homeostasis of Zn metabolism in the pancreas, but also affect the metabolism of Cu. While, ZnSO_4_ had a significant effect on Cu metabolism in the pancreas only when the dose of ZnSO_4_ reached 150 mg kg^−1^. Compared with the same dose of ZnSO_4_, 50 mg kg^−1^ and 100 mg kg^−1^ CS–Zn significantly increased the copper content in the pancreas, indicating that CS–Zn reduced the competitive inhibitory effect of zinc and copper in the process of zinc absorption and transport.

Figure 2B showed the intestinal zinc transporters protein expression of weaned piglets fed dietary CS–Zn or ZnSO_4_. Compared with control group, dietary zinc had a great effect on the protein expressions of ZnT1 and ZIP5 in duodenal mucosa (*p* < 0.05), while the protein expressions of ZnT1 in duodenal mucosa from CS–Zn treatment was enhanced than ZnSO_4_ treatment (*p* < 0.05) (Table 4). The Zn source and added Zn level had significant influence on the protein expression of ZnT1 in duodenal mucosa (*p* < 0.001). The interaction between Zn source and added Zn level affected ZIP5 protein expression (*p* < 0.001). The protein expressions of ZIP5 in duodenal mucosa of piglets fed the diets supplemented with 100 mg kg^−1^ CS–Zn and 150 mg kg^−1^ CS–Zn treatments were higher than piglets from the same level of ZnSO_4_ treatments (*p* < 0.05), whereas there was no difference for that between the 50 mg kg^−1^ CS–Zn and 50 mg kg^−1^ ZnSO_4_ groups (*p* > 0.05). Compared to the other two levels of Zn, the dietary contained 150 mg kg^−1^ Zn, as both CS–Zn and ZnSO_4_ increased the expression of ZnT5 significantly. The protein expressions of ZnT1 and ZIP5 in duodenal mucosa increased linearly with the added Zn level increasing (*p* < 0.001).

Under normal physiological conditions, zinc is a charged bivalent cation, which cannot be transmitted through the cytoplasmic membrane or the endothelium by passive diffusion. Therefore, the transfer of zinc in the body requires a series of proteins to participate in the metabolism. The intestine is the site of excretion and absorption of zinc, which plays a crucial role in maintaining zinc homeostasis. The absorption of zinc in the intestine is shown as unsaturated and saturated kinetics, while the latter process requires a mediated carrier [24]. It is well known that there are two families of zinc transporter protein, the ZIP (SLC39) family and the ZnT (SLC30) family, which play opposite roles in homeostasis. Therefore, the expression of ZnT1 and ZIP5 protein in duodenal mucosa can reflect the ability of the body to absorb and transport Zn as CS–Zn or ZnSO_4_ and, then, deduce the bioavailability of CS–Zn. In the present study, the results showed that CS–Zn or ZnSO_4_ both increased the expression of zinc transporter protein, and the expression of zinc transporter protein increased linearly with supplemental zinc. ZnT1 is distributed on the basolateral membrane of enterocytes; the function of ZnT1 is to transport excess intestinal zinc into extracellular matrix or organelles for the sake of reducing the zinc level of cytoplasm [25]. Increased zinc concentrations significantly elevated intestinal ZnT1 mRNA and protein expression [26]. The current study found that the ZnT1 protein expressions in CS–Zn treatment were more elevated than that in ZnSO_4_ treatment and reached the highest expression in duodenal mucosa when adding 150 mg Zn/kg. Liuzzi et al. (2004) [27] found that the mRNA expression abundance of ZnT1 was decreased in rats with zinc deficiency (<1 mg kg^−1^), while high zinc (180 mg kg^−1^) significantly increased the mRNA expression. ZIP5 is located on the basement membrane of the intestinal epithelial cells, which transports zinc through the basement membrane into the intestinal epithelial cells to maintain the zinc homeostasis. In the absence of zinc, the protein expression of ZIP5 was inhibited, and once the zinc concentration increased rapidly, it was involved in the protein translation process of ZIP5. The current study showed that the Zn source, added Zn level, and their interaction between Zn source and added Zn level had a significant influence on the protein expression of ZIP5 in duodenal mucosa. Weaver et al. (2007) [28] found that the mRNA expression of ZIP5 did not respond to zinc concentration, and zinc ion regulated the translation process of ZIP5 mRNA. Furthermore, the expression of zinc transporter protein in the CS–Zn group was higher than that in the ZnSO_4_ group, indicating that CS–Zn increased the protein expression of ZnT1 and ZIP5, so as to transport zinc to blood, facilitating the zinc transshipment to various tissues and organs.

Relative bioavailability of CS–Zn based on multiple linear regression of zinc content in the liver and pancreas was shown in Table 5. Slope-ratio methodology was used to estimate relative bioavailability of CS–Zn, with the relative bioavailability of ZnSO_4_ standard set at 100%. Using the zinc content in the liver and pancreas as a response parameter, the bioavailability of CS–Zn was 110.9% (*p* < 0.01) and 149.0% (*p* < 0.01), respectively. The zinc content in feces of weaned piglets fed dietary CS–Zn or ZnSO_4_ is shown in Figure 2C. Compared with the same level of Zn as ZnSO_4_, the zinc contents in the feces of weaned piglets fed dietary CS–Zn were reduced by above 30% (*p* < 0.05).

Bioavailability is defined as the proportion of the ingested nutrients being absorbed and available for use or storage. Currently, researchers use the method of determining the accumulation of mineral elements in specific sensitive tissues to assess the bioavailability of mineral element additives. It is well known that the liver and pancreas are sensitive to zinc concentration in a diet [29]; therefore, zinc content in the liver and pancreas are used to estimate zinc bioavailability from different zinc sources. Cao et al. (2000) [30] estimated the bioavailability of polysaccharides–zinc chelate when compared with ZnSO_4_ via multiple linear regression slope-ratio analysis; the results were 110% (liver) and 113% (pancreas), respectively. The bioavailability of the current study was higher for CS–Zn compared with ZnSO_4_. Moreover, fecal zinc content of piglets fed CS–Zn was decreased significantly, which might be associated with the higher bioavailability of CS–Zn in body. Including all concentrations of CS–Zn groups and ZnSO_4_ groups increased zinc concentration in the liver significantly; moreover, there was a linear relationship between them. When the zinc supplementation exceeds the requirement of pigs, the liver zinc concentration is reduced inversely [23], indicating that zinc supplementation was not in excess in the current study. For weaned piglets, the normal digestive function of the pancreas is essential to maintain intestinal health and integrity. Pieper et al. (2015) [31] reported that the activity of intestinal digestive enzymes increased under the high concentration of zinc. Using the pancreas zinc concentration as an index, the present study showed that organic zinc sources had a higher bioavailability than inorganic sources. The results indicated that the pancreas was sensitive to the zinc concentration in a diet. The mechanism of absorption for CS–Zn in the small intestine has not yet been identified. The reason why the bioavailability of CS–Zn in weaned piglets is higher than that of ZnSO_4_ might be related to the absorption and metabolism mechanism of CS–Zn in the small intestine. In brief, CS–Zn harbors its own characters, accelerating the expression of ZnT1 and ZIP5 in the small intestine to increase the concentration of zinc in the liver and pancreas.

## 4. Conclusions

In summary, this study showed a facile and improved method for the synthesis of CS–Zn chelate, and the chelate developed a smaller size and coarser surface compared with CS. There were no lethal effects and pathological symptoms in mice. Weaned piglets fed dietary CS–Zn showed improved growth performance and decreased diarrhea incidence. Moreover, the bioavailability of CS–Zn was higher than that of ZnSO_4_ in piglets, and less fecal zinc was found in the pigs fed dietary CS–Zn. The results indicated that CS–Zn might be a potential substitution of inorganic zinc, reducing zinc excretion and, consequently, affecting the environment. Therefore, the present study might give evidence to further application possibilities of CS–Zn as a novel Zn source in animals and humans. Accordingly, CS–Zn will play a vital role in many fields to offer the chelate function of polysaccharides and metal.

## Figures and Tables

**Table 1:** Composition and nutrient levels of basal diet (as-fed basis).

Ingredients	%	Nutrient Levels ^2^	Content
Corn	62	Digestible energy, MJ/kg	13.75
Soybean meal	16	Crude protein, %	18.12
Extruded soybeans	7	Ether extract, %	3.26
Fish meal	4	Calcium, %	0.93
Whey powder	5	Phosphorus, %	0.79
Bran	2	Zinc, mg/kg	47.30
Salt	0.4		
Calcium hydrogen phosphate	1.1		
Limestone	1.5		
Premix ^1^	1		

^1^ Contained the following per kg of diet: Cu 120 mg (CuSO_4_∙5H_2_O), Fe 400 mg (FeSO_4_∙7H_2_O), Mn 60 mg (MnSO_4_∙4H_2_O), VA 1000 IU, VD 200 IU, VE 60 mg, VB_12_ 10 mg, VB_1_ 1 mg, VB_6_ 2 mg, choline 500 mg, pantothenate acid 10 mg, niacin 35 mg; ^2^ digestible energy was calculated values and others were measured values.

**Table 2:** Effects of dietary Zn source and level on the growth performance of weaned piglets.

Item	Added Zn Level (mg kg^−1^)	ADG (g d^−1^)	ADFI (g d^−1^)	F/G (g g^−1^)
Control	0	231.0 ± 55.40	480.9 ± 13.6	2.08 ± 0.17
ZnSO_4_	50	231.3 ± 17.98 ^Bb^	498.9 ± 20.5 ^Aa^	2.04 ± 0.08 ^Aa^
	100	232.5 ± 46.79 ^Bb^	444.3 ± 23.5 ^Bc^	1.96 ± 0.21 ^Ab^
	150	243.6 ± 25.50 ^Ba^	473.7 ± 5.6 ^Bb^	1.94 ± 0.12 ^Ac^
CS–Zn	50	252.9 ± 42.20 ^Ac^	486.4 ± 26.8 ^Ba^	1.92 ± 0.11 ^Ba^
	100	276.3 ± 19.89 ^Ab^	544.8 ± 24.5 ^Ab^	1.90 ± 0.13 ^Bb^
	150	347.3 ± 34.55 ^Aa^	622.0 ± 19.5 ^Ac^	1.76 ± 0.07 ^Bc^
Zn source	ZnSO_4_	235.8 ± 31.03 ^B^	472.3 ± 25.90 ^B^	1.98 ± 0.46 ^A^
	CS–Zn	292.2 ± 51.93 ^A^	551.1 ± 22.27 ^A^	1.86 ± 0.53 ^B^
Added Zn level (mg kg^−1^)	50	242.1 ± 32.91 ^b^	492.7 ± 13.21 ^b^	1.98 ± 0.63 ^a^
	100	254.4 ± 41.21 ^b^	494.6 ± 26.51 ^b^	1.93 ± 0.34 ^b^
	150	295.5 ± 61.40 ^a^	547.9 ± 35.78 ^a^	1.85 ± 0.85 ^c^
*p*-value	Zn source	<0.001	<0.001	<0.001
	Zn level	0.001	<0.001	<0.001
	Interaction	0.014	<0.001	<0.001

ADG, average daily gain; ADFI, average daily feed intake; F/G, feed/gain ratio. ^A,B^ Means comparison between the factor of Zn source within a column, values with different small letter superscripts mean significant difference (*p* < 0.05). ^a,b,c^ Means comparison between the factor of Zn level within a column, values with different small letter superscripts mean significant difference (*p* < 0.05).

**Table 3:** Effects of dietary Zn source and level on the content of Zn in the liver and pancreas of weaned piglets.

Item	Added Zn Level (mg kg^−1^)	Liver Zn (mg kg^−1^)	Pancreas Zn (mg kg^−1^)
Control	0	70.83 ± 9.29 *	21.02 ± 1.94 *
ZnSO_4_	50	79.57 ± 0.87	38.13 ± 0.88 ^Bc^
	100	99.52 ± 1.38	46.80 ± 4.37 ^Bb^
	150	120.52 ± 13.01	66.04 ± 4.31 ^Ba^
CS–Zn	50	89.44 ± 3.25	43.82 ± 3.76 ^Ac^
	100	106.53 ± 2.95	57.90 ± 4.38 ^Ab^
	150	125.68 ± 4.58	91.65 ± 3.93 ^Aa^
Zn source	ZnSO_4_	99.87 ± 18.76 ^B^	52.57 ± 13.04 ^B^
	CS–Zn	107.22 ± 15.81 ^A^	65.93 ± 21.06 ^A^
Added Zn level (mg kg^−1^)	50	84.51 ± 5.72 ^c^	40.97 ± 4.00 ^c^
	100	103.02 ± 4.32 ^b^	52.97 ± 7.14 ^b^
	150	123.10 ± 9.44 ^a^	77.68 ± 13.94 ^a^
*p*-value	Zn source	0.007	<0.001
	Zn level	<0.001	<0.001
	Interaction	0.732	<0.001
	Linear ^1^	<0.001	<0.001
	Quadratic	0.411	0.127

* Different from all supplemental Zn groups, *p* < 0.05. ^A,B^ Means comparison between the factor of Zn source within a column, values with different small letter superscripts mean significant difference (*p* < 0.05). ^a,b,c^ Means comparison between the factor of Zn level within a column, values with different small letter superscripts mean significant difference (*p* < 0.05). ^1^ Linear effects of added Zn levels.

**Table 4:** Effects of dietary Zn source and level on the protein expressions of ZnT1 and ZIP5 in duodenal mucosa of weaned piglets.

Item	Added Zn Level (mg kg^−1^)	ZnT1	ZIP5
Control	0	3.99 ± 0.30 *	2.99 ± 0.34 *
ZnSO_4_	50	7.84 ± 0.56	7.73 ± 0.78 ^c^
	100	13.50 ± 0.84	12.84 ± 0.39 ^Bb^
	150	19.21 ± 0.78	19.71 ± 0.54 ^Ba^
CS–Zn	50	10.68 ± 0.50	8.42 ± 0.70 ^c^
	100	14.73 ± 0.45	15.59 ± 0.53 ^Ab^
	150	21.15 ± 0.75	27.67 ± 0.66 ^Aa^
Zn source	ZnSO_4_	13.52 ± 4.96 ^B^	13.43 ± 5.23 ^B^
	CS–Zn	15.52 ± 4.60 ^A^	17.23 ± 8.45 ^A^
Added Zn level (mg kg^−1^)	50	9.26 ± 1.63 ^c^	8.08 ± 0.76 ^c^
	100	14.11 ± 0.90 ^b^	14.21 ± 1.56 ^b^
	150	20.18 ± 1.26 ^a^	23.69 ± 4.39 ^a^
*p*-value	Zn source	<0.001	<0.001
	Zn level	<0.001	<0.001
	Interaction	0.150	<0.001
	Linear	<0.001	<0.001
	Quadratic	0.365	0.240

* Different from all supplemental Zn groups, *p* < 0.05. ^A,B^ Means comparison between the factor of Zn source within a column, values with different small letter superscripts mean significant difference (*p* < 0.05). ^a,b,c^ Means comparison between the factor of Zn level within a column, values with different small letter superscripts mean significant difference (*p* < 0.05).

**Table 5:** Relative bioavailability of CS–Zn based on multiple linear regression of zinc content in the liver and pancreas.

Item	Regression Equation	R^2^	*p*	ZnSO_4_	CS–Zn
Liver zinc content	Y = 68.747 + 0.366X1 + 0.330X2	0.899	0.000	100%	110.9%
Pancreas zinc content	Y = 20.186 + 0.447X1 + 0.300X2	0.953	0.000	100%	149.0%

Note: X1 means the amount of Zn as CS–Zn added in the diet, X2 means the amount of Zn as ZnSO_4_ added in the diet.

## Data Availability

The datasets analyzed in the present study are available from the corresponding author on reasonable request.

## Supplementary Materials

The following are available online at https://www.mdpi.com/article/10.3390/ani11092515/s1, Table S1. Analyzed Zn contents in diets for piglets (as fed basis), Table S2. The oral acute toxicity of CS-Zn in mice, Table S3. Effects of dietary Zn source and level on the content of Cu in liver and pancreas of weaned piglets.

## Abbreviation

CS	chitosan
CS–Zn	chitosan–zinc chelated
NRC	National Research Council
AFM	atomic force microscopy
FT-IR	Fourier transform infrared
XRD	X-ray diffraction
AR	acetic acid
ADG	average daily gain
ADFI	average daily feed intake
F/G	feed to gain ratio
ZIP1	Slc 30 A 1 zinc transporter
ZIP5	Slc 39 A 5 zinc transporter
GLM	general linear model
LSD	least significant difference
ANOVA	analysis of variance
