# Supplementary material for: Effects of Zinc Sources and Levels on Growth Performance, Zinc Status, Expressions of Zinc Transporters, and Zinc Bioavailability in Weaned Piglets
Source: Animals (Basel). 2021 Aug 26;11(9):2515. doi: 10.3390/ani11092515 (PMC8470440; doi:10.3390/ani11092515)
Supplement: Supplementary file 1 [file animals-11-02515-s001.zip › animals-1332136-supplementary.pdf]

Supplementary Material

Effects of Zinc Sources and Levels on Growth Performance, Zinc Status, Expressions of Zinc Transporters and Zinc Bioavailability in Weaned Piglets

Xin Ma[1], Mengqi Qian[1], Zhiren Yang[1,2], Tingting Xu[1], Xinyan Han*[1,2]

[1]Key Laboratory of Animal Nutrition and Feed Science in East China, Ministry of Agriculture, College of Animal Science, Zhejiang University, Hangzhou 310058, China

[2] Hainan Institute of Zhejiang University, Yazhou Bay Science and Technology City, Yazhou District, Sanya 572025, China

*Corresponding author.

Xinyan Han

College of Animal Science, Zhejiang University. 866Yuhangtang Road, Hangzhou 310058, P. R. China

Tel: +86-571-88982446

Fax: +86-571-88982650

E-mail: **xyhan@zju.edu.cn**

Table S1. Analyzed Zn contents in diets for piglets (as fed basis).

Zn source	Added Zn (mg kg^{-1})	Analyzed Zn (mg kg^{-1})[a]
$ZnSO_4$	50	104.76
	100	139.88
	150	184.26
Cs-Zn	50	89.52
	100	137.79
	150	206.63

[a]Values based on chemical analysis of triplicate samples of diets.

Table S2. The oral acute toxicity of CS-Zn in mice.

Sex	Dose (g kg^{-1})	number	Initial weight ($\overline{X}$ ± s, g)	Final weight ($\overline{X}$ ± s, g)	Death number	Mortality (%)
Female						
	2.15	5	21.12±0.69	28.93±0.84	0	0
	4.64	5	18.87±0.63	28.62±1.23	1	20
	10.0	5	20.03±0.94	27.00±3.79	2	40
	21.5	5	20.58±1.15	/	5	100
	46.4	5	20.19±1.28	/	5	100
Male						
	2.15	5	20.80±0.82	35.69±3.53	0	0
	4.64	5	19.52±1.11	33.44±2.51	0	0
	10.0	5	20.51±0.87	34.36±2.49	2	40
	21.5	5	20.66±1.22	/	5	100
	46.4	5	21.09±0.51	/	5	100

Table S3. Effects of dietary Zn source and level on the content of Cu in liver and pancreas of weaned piglets.

Item	Added Zn level (mg kg^{-1})	Liver Cu (mg kg^{-1})	Pancreas Cu (mg kg^{-1})
Control	0	21.25±7.01	6.01±0.58
$ZnSO_4$	50	22.74±4.01	6.91±1.36
	100	24.71±9.12	7.69±1.08
	150	21.10±3.66	9.23±1.40
CS-Zn	50	20.32±0.55	9.50±2.11
	100	20.02±8.30	9.60±0.31
	150	21.54±3.56	10.40±1.31
Zn source	$ZnSO_4$	22.60±5.52	8.12±1.59^{B}
	CS-Zn	20.63±4.95	9.86±1.33^{A}
Added Zn level (mg kg^{-1})	50	21.28±2.66	8.20±2.15^{b}
	100	21.90±8.48	8.75±1.23^{ab}
	150	21.32±3.45	9.76±1.43^{a}
p-Value	Zn source	0.269	0.001
	Zn level	0.900	0.40
	Interaction	0.558	0.532
	Linear[1]	0.988	0.048
	Quadratic	0.774	0.727

[A,B] Means comparison between the factor of Zn source within a column, values with different small letter superscripts mean significant difference ($P < 0.05$). [a,b,c] Means comparison between the factor of Zn level within a column, values with different small letter superscripts mean significant difference ($P < 0.05$). [1] Linear effects of added Zn levels.
